# Impact of polymorphisms of the DNA repair gene XRCC1 and their role in the risk of prostate cancer

**DOI:** 10.12669/pjms.312.6653

**Published:** 2015

**Authors:** Haipeng Zhu, Tao Jiu, Dong Wang

**Affiliations:** 1 Haipeng Zhu, Department of Urology Surgery, the Fifth Affiliated Hospital of Zhengzhou University, Zhengzhou, 450052, China; 2 Tao Jiu, Department of Urology Surgery, the Fifth Affiliated Hospital of Zhengzhou University, Zhengzhou, 450052, China; 3 Dong Wang, Department of Urology Surgery, the Fifth Affiliated Hospital of Zhengzhou University, Zhengzhou, 450052, China

**Keywords:** DNA repair-related genes, Prostate cancer, Susceptibility, XRCC1

## Abstract

**Objective:**

We conducted a case-control study to examine the role of XRCC1 codons 194 (Arg>Trp), 280 (Arg>His) and 399 (Arg>Gln) polymorphisms in the risk of prostate cancer.

**Methods:**

This study included 572 consecutive primary prostate cancer patients and 572 controls between January 2011 and January 2014. The polymerase chain reaction-restriction fragment length polymorphism (PCR-RFLP) was performed to detect XRCC1 codons 194 (Arg>Trp), 280 (Arg>His) and 399 (Arg>Gln) polymorphisms.

**Results:**

Compared with the control subjects, the prostate cancer cases had a habit of cigarette smoking (χ^2^=18.13, *P*<0.001) and a family history of cancer (χ^2^=25.23, *P*<0.001). Conditional logistic regression analysis showed that the subjects carrying Trp/Trp genotype were more likely to greatly increase the prostate cancer when compared with Arg/Arg genotype, and the adjusted OR was 2.04(1.24-3.41). We did not find significant association between XRCC1 194 (Arg>Trp) polymorphism and clinical stage and Gleason score of prostate cancer (*P*>0.05).

**Conclusion:**

Our results show an increased risk for prostate cancer in individuals with XRCC1 194 (Arg>Trp) polymorphism, and a significant interaction between XRCC1 194 (Arg>Trp) polymorphism and tobacco smoking, alcohol drinking and family history of cancer.

## INTRODUCTION

Prostate cancer is the second most common cancer in men, and the fourth overall. It is estimated that there were 1.1 million men in 2012 worldwide, accounting for 15% of the cancers diagnoses in men.^1^ It is well known that prostate cancer is caused by various factors, including environmental and genetic factors.^2^-^4^

Previous studies showed that genetic polymorphisms in DNA repair genes can lead to differential capacity to repair DNA damage, which may cause genetic instability and carcinogenesis.^5^ In mammalian cells four different DNA repair mechanisms have been identified: base excision repair (BER), nucleotide excision repair (NER), double-strand break repair and mismatch repair.^6^,^7^ These DNA repair pathways can influence the maintenance of genomic integrity and modulation of repair capacity in response to DNA damage and thus susceptibility to cancers.^8^

XRCC1 (X-ray repair cross-complementing group1) gene is located on chromosome 19q13.2 which codes for a 633 amino acid residue protein that acts as scaffolding protein. XRCC1 protein functions in a complex with many other components to facilitate BER and single-strand break-repair processes.^9^ Several SNPs in XRCC1 have been identified, all of which affect the coding region of XRCC1. These coding polymorphisms were detected at codons 194 (Arg>Trp), 280 (Arg>His) and 399 (Arg>Gln) in different cancers including prostate cancer, but the results are inconsistent.^10^-^13^ These non- conservative amino acid changes may alter XRCC1 function and may have an impact on individual susceptibility to prostate cancer. Therefore, in this study, we conducted a case-control study to examine the role of XRCC1 codons 194 (Arg>Trp), 280 (Arg>His) and 399 (Arg>Gln) polymorphisms in the risk of prostate cancer.

## METHODS

### Patients

This study included 572 consecutive primary prostate cancer patients. All the prostate cancer patients were newly diagnosed and histopathologically confirmed primary prostate cancer from the Fifth Affiliated Hospital of Zhengzhou University between January 2011 and January 2014. Tumor types and stages were determined by two pathologists. The cases who had not received any chemotherapy or radiotherapy were selected into our study. Total 572 control subjects were randomly selected from individuals who came to undergo a routine health examination during the same period. All the control subjects were found to be without cancers. The controls were matched with one case by age at enrollment (within ±5 years). Demographic and lifestyle characteristics of cases and controls were taken on a predesigned questionnaire. All the patients and controls agreed to sign an informed consent before entering the study.

The collection and use of tumor and blood samples for this study were previously approved by ethnics committee of the Fifth Affiliated Hospital of Zhengzhou University.

### DNA extraction and genotype analysis

Each subject was asked to provide 5-ml venous blood sample for DNA preparation. 0.5mg/ml EDTA was taken for anticoagulant of blood, and the blood was stored in -20ºC until use. Genomic DNA was extracted from a peripheral blood with TIANamp Blood DNA Kit (Tiangen, Beijing, China) according to the manufacturer’s instructions. The polymerase chain reaction-restriction fragment length polymorphism (PCR-RFLP) was performed to detect XRCC1 codons 194 (Arg>Trp), 280 (Arg>His) and 399 (Arg>Gln) polymorphisms. The primers of XRCC1 codons 194 (Arg>Trp), 280 (Arg>His) and 399 (Arg>Gln) were designed using Sequenom Assay Design 3.1 software (Sequenom, San Diego, CA). Briefly PCR was carried out in a final volume of 25 μL containing 50 ng genomic DNA template, 1×PCR buffer with 2 mM MgCl2, 0.5 μM of each primer, 50 μM dNTPs and 0.5 U DNA polymerase. For PCR amplification, the standard program was used as follows: one initial denaturation step at 94°C for 7 minutes, followed by 35 denaturation cycles of 1min at 94°C, 1min of annealing at 60°C, and one minutes of extension at 72°C, followed by a final elongation cycle at 72°C for 10 min.

### Statistical analysis

Differences in the distributions of demographic, lifestyle and clinical characteristics and genotypes of XRCC1 codons 194 (Arg>Trp), 280 (Arg>His) and 399 (Arg>Gln) between cases and controls were calculated by χ^2^-test. The goodness-of-fit χ^2^-test was used to verify whether the genotype distributions of XRCC1 codons 194 (Arg>Trp), 280 (Arg>His) and 399 (Arg>Gln) were in Hardy–Weinberg equilibrium. The associations between XRCC1 codons 194 (Arg>Trp), 280 (Arg>His) and 399 (Arg>Gln) gene polymorphisms and risk of prostate cancer were assessed by conditional logistic regression with the adjustment for potential confounding factors, and the assessed results were expressed with odds ratio (OR) and 95% confidence intervals (CI). Gene-environmental interaction was evaluated by conditional logistic regression. All *P*-values were two sided, and statistically significance was set at *P*-value less than 0.05. All statistical analyses were performed using the STATA version 10.0 software.

## RESULTS

The demographic and clinical characteristics of included prostate cancer cases and controls are shown in Table-I. We did not find significant difference between included prostate cancer cases and controls in terms of age and drinking status (*P*>0.05). Compared with the control subjects, the prostate cancer cases had a habit of cigarette smoking (χ^2^=18.13, *P*<0.001) and a family history of cancer (χ^2^=25.23, *P*<0.001). Of the 572 prostate cancer patients, 277 (48.43%) patients had advanced prostate cancer, 295 (51.57%) had localized cancer, 204(35.66%) had Gleason score <7, and 368(64.34%) had Gleason score≥7.

**Table-I:** Characteristics of the prostate cancer cases and controls.

Characteristics	Cases, N=572	%	Controls, N=572	%	χ^2^ value	P value
Age, years (mean±SD)	70.7±9.5		70.4±9.2			
<70	301	52.62	304	53.15		
≥70	271	47.38	268	46.85	0.03	0.86
Cigarette smoking						
Never	245	42.83	317	55.42		
Ever	327	57.17	255	44.58	18.13	<0.001
Alcohol drinking						
Never	244	42.66	255	44.58		
Ever	328	57.34	317	55.42	0.43	0.51
Family history of cancer						
Never	483	84.44	536	93.71		
Ever	89	15.56	36	6.29	25.23	<0.001
Clinical stage						
Localized	295	51.57				
Advanced	277	48.43				
Gleason score						
<7	204	35.66				
≥7	368	64.34				

The genotype distributions of XRCC1 codons 194 (Arg>Trp), 280 (Arg>His) and 399 (Arg>Gln) in cases and controls are shown in Table-II. By goodness-of-fit χ^2^-test, we found that genotype distributions of XRCC1 codons 194 (Arg>Trp) and 399 (Arg>Gln) in controls conforms to Hardy-Weinberg equilibrium, while 280 (Arg>His) was not. Conditional logistic regression analysis showed that the subjects carrying Trp/Trp genotype were more likely to greatly increase the prostate cancer when compared with Arg/Arg genotype, and the adjusted OR was 2.04(1.24-3.41). However, no association was found between polymorphisms in XRCC1 codons 194 (Arg>Trp) and 399 (Arg>Gln) and risk of prostate cancer (*P*>0.05).

**Table-II:** Genotype frequencies of XRCC1 codons 194 (Arg>Trp), 280 (Arg>His) and 399 (Arg>Gln) polymorphisms in prostate cancer cases and controls.

Genotype	Cases	%	Controls	%	P value for Hardy-Weinberg equilibrium	Adjusted OR (95%CI)^1^	P value
XRCC1 194 (Arg>Trp)							
Arg/Arg	310	54.2	340	59.44		1.0(Ref.)	-
Arg/Trp	208	36.36	203	35.49		1.12(0.87-1.45)	0.35
Trp/Trp	54	9.44	29	5.07	0.85	2.04(1.24-3.41)	<0.003
XRCC1 280 (Arg>His)							
Arg/Arg	380	66.43	394	68.88		1.0(Ref.)	-
Arg/His	120	20.98	116	20.28		1.07(0.79-1.45)	0.64
His/His	73	12.76	62	10.84	<0.001	1.22(0.83-1.79)	0.29
XRCC1 399 (Arg>Gln)							
Arg/Arg	249	43.53	276	48.25		1.0(Ref.)	-
Arg/Gln	245	42.83	243	42.48		1.07(0.83-1.37)	0.61
Gln/Gln	78	13.64	53	9.27	0.96	1.32(0.87-2.01)	0.18

1 Adjusted for age, cigarette smoking, alcohol drinking and family history of cancer in conditional logistic regression model.

Stratification analyses of age, tobacco smoking, alcohol drinking and family history of cancer with XRCC1 194 (Arg>Trp) polymorphism are shown in Table-III. Compared with Arg/Arg genotype, individuals with Arg/Trp+Trp/Trp genotype had a significantly increased risk of prostate cancer in tobacco smokers, alcohol drinkers and individuals who had no family history of cancer, with adjusted OR(95%CI) of 1.57(1.11-2.23), 1.47(1.06-2.04) and 2.95(1.10-8.78).

**Table-III:** Stratification analyses between XRCC1 194 (Arg>Trp)polymorphism and risk of prostate cancer.

Variables	Cases / Controls	Genotypes, Cases				Controls				Adjusted OR(95%CI)	P value
		Arg/Arg		Arg/Trp + Trp/Trp		Arg/Arg		Arg/Trp + Trp/Trp		Arg/Trp + Trp/Trp versus Arg/Arg
		n	%	n	%	n	%	n	%	
*Age, years*											
<70	301/304	157	50.65	144	54.96	178	52.35	126	54.31	1.30(0.93-1.81)	0.11
≥70	271/268	153	49.35	118	45.04	162	47.65	106	45.69	1.18(0.82-1.69)	0.35
*Smoking status*																					
Never	245/317	138	44.52	107	40.84	178	52.35	139	59.91	0.99(0.70-1.41)	0.97
Ever	327/255	172	55.48	155	59.16	162	47.65	93	40.09	1.57(1.11-2.23)	0.008
*Drinking status*																					
Never	244/255	136	43.87	108	41.22	142	41.76	113	48.71	1.00(0.69-1.44)	0.99
Ever	328/317	174	56.13	154	58.78	198	58.24	119	51.29	1.47(1.06-2.04)	0.02
*Family history of cancer*																				
Never	483/536	258	83.23	225	85.88	311	91.47	225	96.98	1.21(0.93-1.56)	0.14
Ever	89/36	52	16.77	37	14.12	29	8.53	7	3.02	2.95(1.10-8.78)	0.02

1. Adjusted for age, cigarette smoking, alcohol drinking and family history of cancer in conditional logistic regression model.

## DISCUSSION

In this case-control study, we investigated that the role of XRCC1 codons 194 (Arg>Trp), 280 (Arg>His) and 399 (Arg>Gln) polymorphism in the risk of prostate cancer, and the gene-environmental interaction on the development of prostate cancer.

Since there is increasing evidence that genetic variation leads to different DNA repair capacities in the human population, several common polymorphisms in BER pathway can play a role in individuals’ genetic susceptibility to cancer.^14^ Mutations in XRCC1 gene may play a role in decreasing or losing of its DNA repair capacity and confering the variation in susceptibility to diverse malignant tumors among individuals. It is reported that there were more than 300 SNPs in XRCC1 gene, and XRCC1 Arg194Trp and XRCC1 Arg399Gln polymorphisms are the most common studies SNPs.^8^,^14^ Many studies have indicated that the XRCC1 Arg194Trp polymorphism may influence the development of several kinds of cancer, including glioma and thyroid cancer,^15^,^16^ but several meta-analysis showed that no association between XRCC1 Arg194Trp polymorphism and risk of head and neck cancer, hepatocellular carcinoma, lung cancer and head and neck cancer.^16^-^18^

For prostate cancer, several epidemiological studies investigated the association between XRCC1 polymorphisms and susceptibility to prostate cancer.^19^-^21^ However, the results of these studies are inconsistent. Xu et al. investigate the correlation between XRCC1 codons 194 (Arg>Trp), 280 (Arg>His) and 399 (Arg>Gln) polymorphisms and prostate cancer risk, and they suggested that XRCC1 Arg280His and Arg399Gln variant genotypes have a role in the development of prostate cancer, and have interaction with heavy tobacco smoking.^19^ Another study conducted in a north Indian population, and reported that Gln/Gln genotype of XRCC1 codons 399 (Arg>Gln) greatly increased the risk of prostate cancer.^21^ However, Dhillon et al. did not find significant association between XRCC1 polymorphisms and prostate cancer risk.^20^ Recent two meta-analysis showed that XRCC1 codons 194 (Arg>Trp) and 399 (Arg>Gln) were not significantly associated with risk of prostate cancer.^22^,^23^ The discrepancies of the finding from previous epidemiological studies could be elucidate by differences in populations, source of prostate cancer patients or cancers, lifestyles of populations, sample size study design and also by chance.

Our study found that XRCC1 194 (Arg>Trp) polymorphism has interaction with tobacco smoking, alcohol drinking and family history of cancer, which indicated that a significantly gene-environment interaction was shown in the risk of prostate cancer. A previous study showed that XRCC1 Arg399Gln variant genotypes was associated with a heavy increased risk of prostate cancer in heavy smokers,^19^ and their results are in line with ours.

There were three limitations in this study. First, the cases and controls were selected from one hospital, and thus selection bias may be existed in this study. Second, we only investigated association between XRCC1 and risk of prostate cancer, and other DNA repaired genes may have interaction with XRCC1. Third, the sample size of this study is relatively small, and this small sample size may limit the statistical power to find the difference between groups. Therefore, large sample size studies with more ethnicities are greatly needed to confirm our results.

In conclusion, our results show an increased risk for prostate cancer in individuals with XRCC1 194 (Arg>Trp) polymorphism, and a significant interaction between XRCC1 194 (Arg>Trp) polymorphism and tobacco smoking, alcohol drinking and family history of cancer.
